# HMGB1: A Promising Therapeutic Target for Prostate Cancer

**DOI:** 10.1155/2013/157103

**Published:** 2013-05-12

**Authors:** Munirathinam Gnanasekar, Ramaswamy Kalyanasundaram, Guoxing Zheng, Aoshuang Chen, Maarten C. Bosland, André Kajdacsy-Balla

**Affiliations:** ^1^Department of Biomedical Sciences, College of Medicine, University of Illinois, 1601 Parkview Avenue, Rockford, IL 61107, USA; ^2^Department of Pathology, University of Illinois at Chicago, Chicago, IL 60612, USA

## Abstract

High mobility group box 1 (HMGB1) was originally discovered as a chromatin-binding protein several decades ago. It is now increasingly evident that HMGB1 plays a major role in several disease conditions such as atherosclerosis, diabetes, arthritis, sepsis, and cancer. It is intriguing how deregulation of HMGB1 can result in a myriad of disease conditions. Interestingly, HMGB1 is involved in cell proliferation, angiogenesis, and metastasis during cancer progression. Furthermore, HMGB1 has been demonstrated to exert intracellular and extracellular functions, activating key oncogenic signaling pathways. This paper focuses on the role of HMGB1 in prostate cancer development and highlights the potential of HMGB1 to serve as a key target for prostate cancer treatment.

## 1. Introduction

Current treatment methods for prostate cancer (PCa) such as radical prostatectomy, chemotherapy, radiation therapy, or hormonal therapy are used to effectively manage this disease. However, majority of patients undergoing androgen deprivation therapy develop castration resistant PCa [1]. Hence, there is a great interest in understanding the molecular events that are critical for the development of this disease. If characterized, the genes that play a crucial role in PCa progression or hormone resistance PCa will result in development of novel strategies for treating PCa. Recent evidences strongly suggest that high mobility group box 1 (HMGB1) plays a pivotal role in the development of several cancer types including PCa [2–4]. It is found to be associated with all the hallmarks of cancer development such as cell proliferation, anchorage-independent growth, angiogenesis, migration, and invasion [3].

 HMGB1 is a DNA binding protein involved in DNA replication and DNA repair process [5]. Outside the cell, it functions as a proinflammatory cytokine [6]. The extracellular receptors of HMGB1 include RAGE and TLR4, with RAGE being implicated as a major receptor for HMGB1 in tumor development. Deregulation of HMGB1 has been shown to be associated with several inflammation associated diseases such as atherosclerosis [7, 8], arthritis [9], and sepsis [10]. Moreover, HMGB1 is also shown to promote tumorigenesis by inducing inflammation [11, 12]. Inflammation is one of the key risk factors implicated in prostate carcinogenesis [13–15]. Based on the recent published evidences, we highlight and speculate on the role of HMGB1 in PCa development and the potential strategies to target HMGB1 for PCa treatment.

## 2. HMGB1 Expression in Prostate Cancer Cells: Preclinical and Clinical Samples

HMGB1 is known to be consistently overexpressed in cancer cells compared to normal cell types [3, 16–18]. Similarly, HMGB1 is also reported to be highly expressed in PCa cells [4, 19, 20]. Interestingly, androgen deprivation resulted in the secretion of HMGB1 in prostatic stromal cells and found to be associated with metastatic PCa [21]. This finding support the notion that androgen deprivation therapy may upregulate the expression of HMGB1 leading to either hormone resistance or metastatic disease. 

Studies conducted by He et al. [22] employing transgenic adenocarcinoma mouse prostate (TRAMP) model demonstrated that HMGB1 promotes invasive carcinoma in this experimental setting. Furthermore, their study also showed that HMGB1 is released in the serum during tumor progression correlating with severity of disease pathology. Previous studies have shown that serum HMGB1 can serve as a biomarker for variety of cancers such as pancreatic ductal adenocarcinoma [23], colorectal carcinoma [24], malignant mesothelioma [25], canine lymphoma [26], non-small-cell lung cancer [27, 28], gastric cancer [29], and hepatocellular carcinoma [30]. However, a study conducted by Mengus et al. [31] to determine the circulating levels of cytokines in early stage prostate cancer (1 to 2c) showed that HMGB1 levels were not found to be significant when compared to control benign hyperplastic prostate (BPH) samples. These results combined with serum levels of HMGB1 in the TRAMP mouse PCa model may suggest that HMGB1 be a marker for advanced stages of PCa. 

Expression of HMGB1 in clinical samples was first reported by Kuniyasu et al. [21] in a pilot study where they found that HMGB1 is expressed in tumor (27%) and stromal cells (63%) of metastatic patients. Interestingly, they also observed that HMGB1 was not expressed (0%) in tumors of nonmetastatic cases, while only 11% of patients with nonmetastases expressed HMGB1 in stromal cells. In subsequent study, Ishiguro et al. [19] using real-time quantitative PCR showed that HMGB1 and its cognate receptor, RAGE, are significantly expressed in primary PCa and refractory samples compared to normal control prostate samples. More recently, Li et al. [20] determined the correlation pattern of HMGB1 expression with clinical characteristics of PCa. Their findings showed that about 60% (101/168) of PCa cases were positive for HMGB1 expression. Specifically, this study revealed that HMGB1 expression correlated with stage of cancer (pT), Gleason grade, preoperative prostate specific antigen, biochemical recurrence, and poor survival rates. Thus, these *in vitro*, preclinical and clinical evidences strongly point that HMGB1 may have a pivotal role in the progression of PCa.

## 3. HMGB1 Interacting Genes/Proteins in Prostate Cancer

HMGB1 has been reported to transactivate sex steroid hormone receptors such as androgen receptor, mineralocorticoid receptor, progesterone receptor, and glucocorticoid receptor [32, 33]. In PCa, transactivation of androgen receptor (AR) by HMGB1 [33] may have clinical significance. AR is a crucial gene required for PCa survival and PCa progression [34, 35]. In addition, AR activation is also known to play a major role in the development of androgen-independent PCa [34–36]. Activation or expression of AR is shown to be regulated by many signaling pathways [36–38]. Our recent publication showed that targeting receptor for advanced glycation end products (RAGEs) downregulated the expression of prostate specific antigen (PSA), the downstream target gene of AR [39], suggesting that RAGE may have a role in the regulation of AR in PCa cells. Interestingly, previous study by Ishiguro et al. [19] showed that both RAGE and HMGB1 are coexpressed in PCa samples and suggested that they may have cooperative role in the progression of PCa. Thus, HMGB1 may regulate AR either by acting as co-activator of AR or indirectly associating with RAGE signaling in prostate oncogenesis.

HGMB1 and RAGE have been shown to interact in many types of tumor cells but not in normal cells [40]. In extracellular milieu, HMGB1 may interact with RAGE receptor in PCa cells. This notion is supported by our recent work [39], which showed that silencing RAGE expression by RNAi approach abrogated the cell proliferative effects of extracellular recombinant HMGB1 on PCa cells. Interestingly, HMGB1 is also shown to enhance DNA binding activity of ETS transcription factor in regulating peroxiredoxin-1 and -5 expression in combating oxidative stress in PCa cells [41]. That HMGB1 directly interact with ETS to enhance its target gene transcriptional activity may have significant implications in PCa disease progression, as ETS is known to play a major role in PCa progression, androgen independence, and metastatic progression [42–46]. Given the recent findings that HMGB1 can facilitate gene recombination [5, 47, 48] and the fact that frequent gene rearrangements of ETS derived transcription factors are detected in PCa [42, 49], the possibility of ETS gene recombination driven by HMGB1 may favor promotion of aggressive PCa. 

## 4. Possible HMGB1 and Inflammation Link in PCa

**Figure 1:**
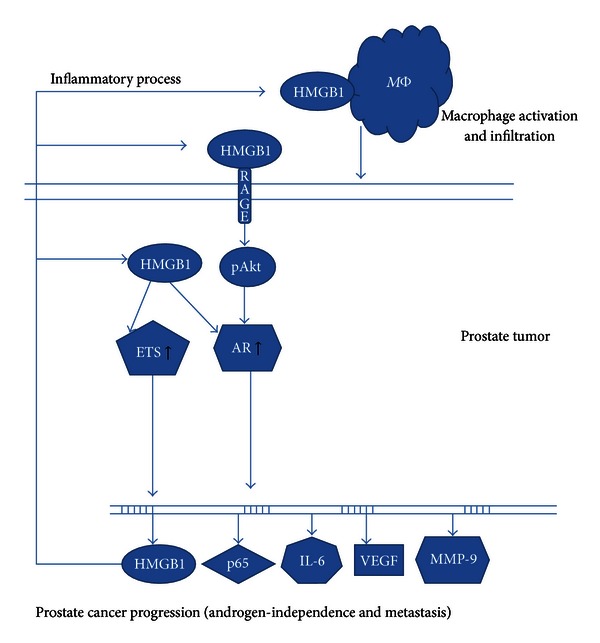
Proposed model of HMGB1 mediated prostate cancer progression.

Risk factors for PCa include genetic factors, hormonal changes, chronic inflammation, and dietary differences [14, 50]. Among these etiological factors, there is growing evidence for the role of inflammation in the prostate carcinogenesis [15, 51, 52], in particular chronic inflammation [14, 15, 53]. The role of inflammation in the prostate carcinogenesis is now widely accepted [15, 51, 54]. HMGB1-RAGE axis plays a major role in inflammation induced carcinogenesis [55, 56]. Evidence for the role of HMGB1 in PCa inflammation can be inferred from a recent study by He et al. [22] using TRAMP animal model of PCa. In this study, they showed that targeting HMGB1 disrupts tumor progression by inhibiting activation of T-cells and reducing infiltration of macrophages, which are considered to be key inflammatory cells in promoting variety of cancers including PCa [57–60]. Thus, inflammation may be one mechanism by which HMGB1 may accelerate PCa as depicted in Figure 1. In our recent work [61], we also showed that HMGB1 is one of the target inflammatory gene for 18-alpha glycyrrhetinic acid in PCa cells. Our study thus suggests that inflammation associated genes such as HMGB1 may play a vital role in the multistep process of PCa development.

## 5. Can HMGB1 Be a Viable Target for PCa Treatment?

HMGB1 is suggested to be a potential target gene for various diseases such as atherosclerosis, inflammation, sepsis, and arthritis [7, 8]. Accumulating evidences suggest that HMGB1 can also serve as a target for various cancer types including PCa [4, 62–65]. Some potential HMGB1 targeting strategies are highlighted here.

## 6. Gene Targeting

Antisense and RNA interference (RNAi) technology are most commonly used strategies to eliminate or silence the expression of target genes [66]. These technologies are also used as a tool to study gene function in cancer cells [67]. Interestingly, several preclinical and early clinical trials have shown great promise of these strategies for use in PCa treatment [68–72].

Studies performed by Kuniyasu et al. [21] showed that antisense targeting of HMGB1 in PC-3 cells significantly inhibited the invasive potential of these cells *in vitro*. In our recent work [4], we showed that targeting HMGB1 by RNAi resulted in the inhibition of PCa cell proliferation and apoptotic elimination of PCa cells. Furthermore, our additional work also revealed that targeting RAGE by RNAi prevented HMGB1-mediated cell proliferation of PCa cells and reduction of HMGB1 levels in the RAGE RNAi transfected cells. This also led to growth inhibition of androgen-dependent and -independent prostate tumor in nude mice model. Targeting HMGB1 by RNAi is also shown to inhibit osseous metastasis of PC cells in an experimental metastases model [73]. Thus antisense and RNAi strategies represent promising methods to target HMGB1 expression to achieve therapeutic effects against PCa. 

## 7. Antibody (Ab) Based Targeting

Ab based treatment approach is a promising strategy to target PCa. For example, anti-VEGF antibody treatment has yielded encouraging results in clinical trials [74]. However, the anti-VEGF Ab may primarily target angiogenesis process of tumor growth. In this case, HMGB1 may be a desirable target for prostate PCa, as it is involved in cell proliferation, apoptosis regulation, angiogenesis, and metastases [3]. In support of this, studies by He et al. [22] showed that administration of anti-HMGB1 significantly inhibited the prostate tumor progression in TRAMP mouse PCa model. In another study [75], anti-HMGB1 was shown to inhibit HMGB1-enforced angiogenic process of colon cancer cells. Furthermore in a malignant mesothelioma study, targeting HMGB1 by mAb also showed to effectively inhibit the matrigel invasion of malignant mesothelial cells *in vitro*, hinder the tumor growth, and extend the survival of nude mice. The potential of anti-HMGB1 to inhibit colon cancer development was also recently demonstrated [76, 77]. Collectively these studies attest to the therapeutic utility of anti-HMGB1 antibody for cancer treatment in general, which can also be developed for PCa treatment. 

## 8. Use of Natural Products

Naturally occurring agents such as glycyrrhizin, glycyrrhetinic acid, ethyl pyruvate, and green tea phenols have been shown to target HMGB1 expression in variety of cell/disease models [61, 78–84]. Specifically, we showed that 18-alpha glycyrrhetinic acid, a derivative of glycyrrhizin that is abundantly present in the licorice root, can decrease HMGB1 gene expression and result in the therapeutic effects in PCa cells [61]. Other natural compounds known to target HMGB1 include some cholinergic agonists [85, 86], thrombomodulin [87–89], and low molecular-weight heparin [90]. All these natural agents could be potentially tested against PCa cells/tumors that display high levels of HMGB1 expression. 

## 9. Other Potential HMGB1 Targeting Therapies

Recent studies advocate that peptide (A-Box) derived from HMGB1 can be used to effectively antagonize the functions of HMGB1 [91, 92]. This A-Box has been shown to downregulate the inflammatory activity of HMGB1 [91]. It has also been postulated to inhibit tumor angiogenesis by disrupting HMGB1-RAGE signaling [93]. Although, the anti-cancer potential of A-Box is yet to be evaluated against PCa, it may offer novel treatment strategy for PCa given the fact that A-Box can be used as gene delivery agent [94, 95].

Targeting extracellular HMGB1 represents another strategy to combat PCa as we recently showed that addition of rHMGB1 promotes PCa cell proliferation [39]. Secreted HMGB1 can be neutralized by administering soluble RAGE as discussed for controlling atherosclerosis [8] and inflammation [96]. Interestingly, the endogenous levels of soluble RAGE are downregulated in patients with liver cancer [97], colorectal adenoma [98], pancreatic cancer [99, 100], lung cancer [101], and breast carcinoma [102]. However, the status of soluble RAGE in PCa patients is not yet known and warrants further investigation for PCa treatment. 

Developing HMGB1 as a vaccine for PCa is a feasible immunotherapeutic strategy as the previous study strongly support that peptides derived from HMGB1 engrafted in liposomes induced potent antigen-specific and tumor specific immunity against B16-OVA melanoma model [103]. A follow-up study also demonstrated that HMGB1 antigenic peptides can act as an adjuvant for subunit cancer vaccines [104]. These HMGB1 derived peptides could also be tested as adjuvant for enhancing the efficacy of PCa vaccines that are currently under development [105–107].

## 10. Concluding Remarks

The evidence for role of HMGB1 in cancer progression is now rapidly accumulating. Studies from others and our group suggest that HMGB1 may also have a prominent role in PCa development. The interaction of HMGB1 with AR, RAGE, and ETS point to a central role of HMGB1 in PCa progression. Clinical studies also support that HMGB1 is overexpressed in PCa patients and may serve as a novel prognostic marker for BCR-free survival for prostate cancer patients after undergoing radical prostatectomy. Importantly, several preclinical studies highlighted in this paper suggest that HMGB1 can be targeted by variety of approaches, which may ultimately lead to the development of effective therapy for PCa patients.
